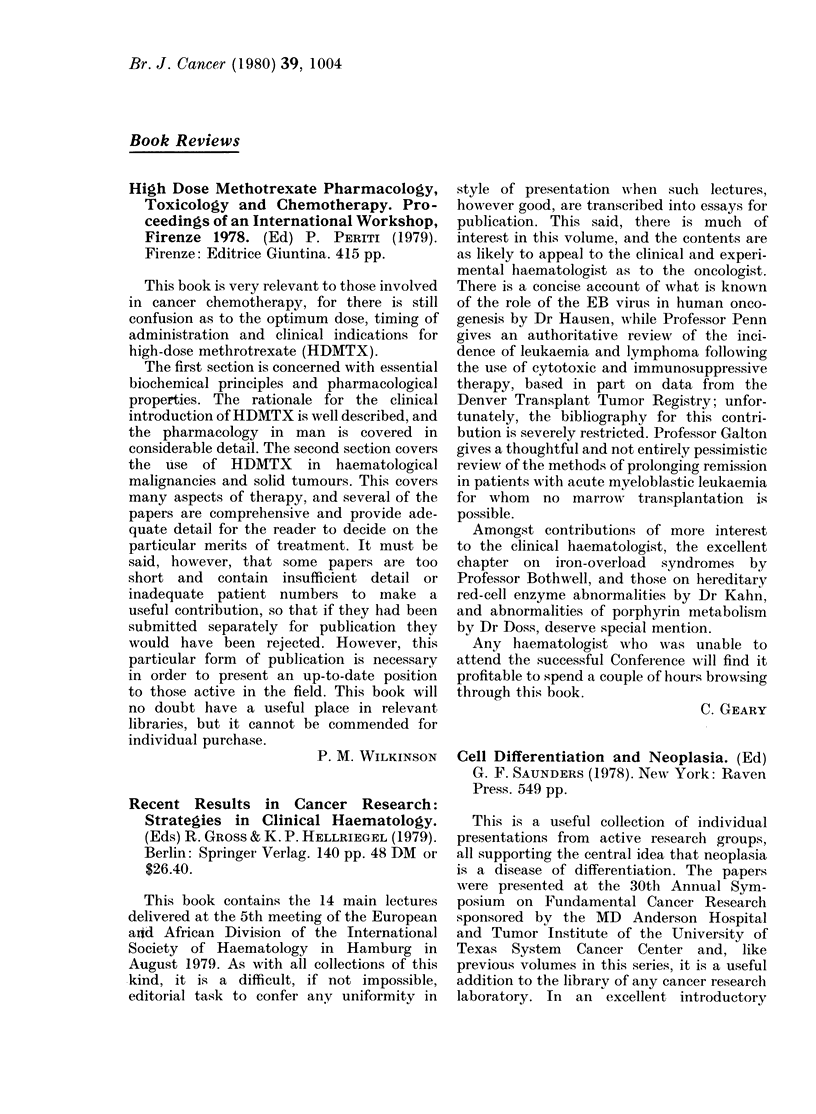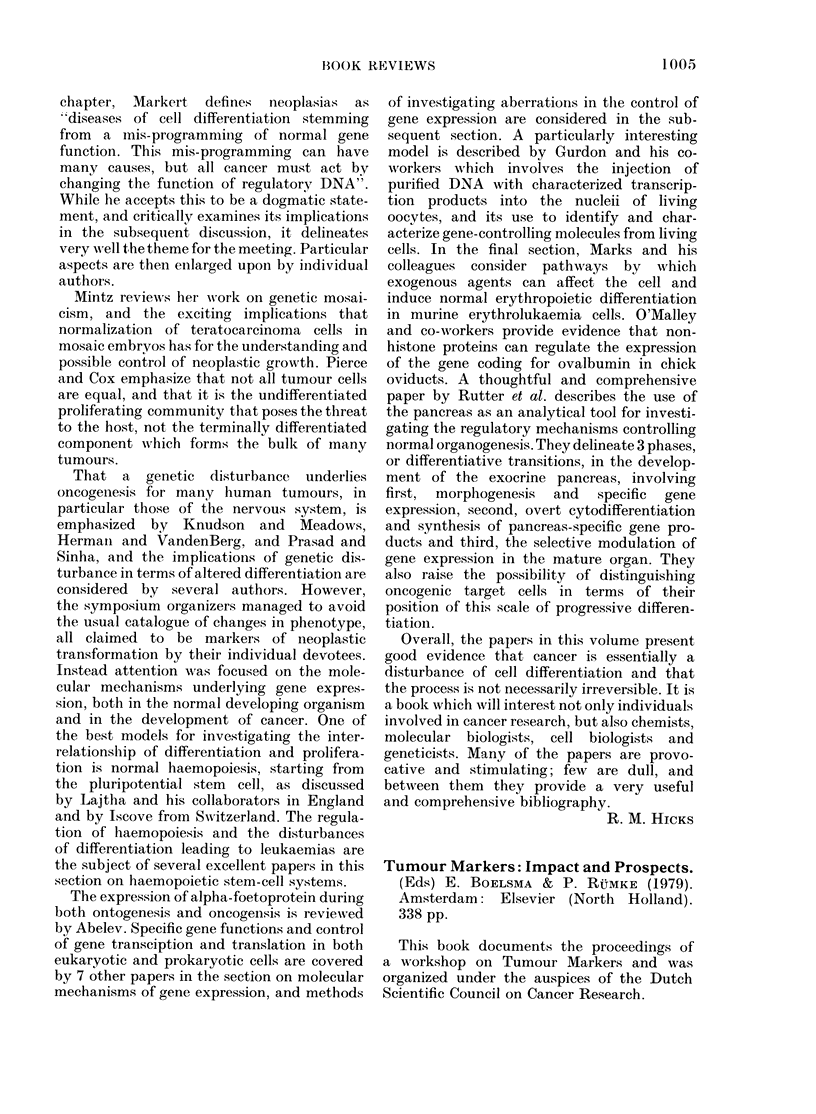# Cell Differentiation and Neoplasia

**Published:** 1980-06

**Authors:** R. M. Hicks


**Cell Differentiation and Neoplasia.** (Ed) G. F. SAUNDERS (1978). New York: Raven Press. 549 pp.

This is a useful collection of individual presentations from active research groups, all supporting the central idea that neoplasia is a disease of differentiation. The papers were presented at the 30th Annual Symposium on Fundamental Cancer Research sponsored by the MD Anderson Hospital and Tumor Institute of the University of Texas System Cancer Center and, like previous volumes in this series, it is a useful addition to the library of any cancer research laboratory. In an excellent introductory chapter, Markert defines neoplasias as "diseases of cell differentiation stemming from a mis-programming of normal gene function. This mis-programming can have many causes, but all cancer must act by changing the function of regulatory DNA". While he accepts this to be a dogmatic statement, and critically examines its implications in the subsequent discussion, it delineates very well the theme for the meeting. Particular aspects are then enlarged upon by individual authors.

Mintz reviews her work on genetic mosaicism, and the exciting implications that normalization of teratocarcinoma cells in mosaic embryos has for the understanding and possible control of neoplastic growth. Pierce and Cox emphasize that not all tumour cells are equal, and that it is the undifferentiated proliferating community that poses the threat to the host, not the terminally differentiated component which forms the bulk of many tumours.

That a genetic disturbance underlies oncogenesis for many human tumours, in particular those of the nervous system, is emphasized by Knudson and Meadows, Herman and VandenBerg, and Prasad and Sinha, and the implications of genetic disturbance in terms of altered differentiation are considered by several authors. However, the symposium organizers managed to avoid the usual catalogue of changes in phenotype, all claimed to be markers of neoplastic transformation by their individual devotees. Instead attention was focused on the molecular mechanisms underlying gene expression, both in the normal developing organism and in the development of cancer. One of the best models for investigating the interrelationship of differentiation and proliferation is normal haemopoiesis, starting from the pluripotential stem cell, as discussed by Lajtha and his collaborators in England and by Iscove from Switzerland. The regulation of haemopoiesis and the disturbances of differentiation leading to leukaemias are the subject of several excellent papers in this section on haemopoietic stem-cell systems.

The expression of alpha-foetoprotein during both ontogenesis and oncogensis is reviewed by Abelev. Specific gene functions and control of gene transciption and translation in both eukaryotic and prokaryotic cells are covered by 7 other papers in the section on molecular mechanisms of gene expression, and methods of investigating aberrations in the control of gene expression are considered in the subsequent section. A particularly interesting model is described by Gurdon and his co-workers which involves the injection of purified DNA with characterized transcription products into the nucleii of living oocytes, and its use to identify and characterize gene-controlling molecules from living cells. In the final section, Marks and his colleagues consider pathways by which exogenous agents can affect the cell and induce normal erythropoietic differentiation in murine erythrolukaemia cells. O'Malley and co-workers provide evidence that non-histone proteins can regulate the expression of the gene coding for ovalbumin in chick oviducts. A thoughtful and comprehensive paper by Rutter *et al.* describes the use of the pancreas as an analytical tool for investigating the regulatory mechanisms controlling normal organogenesis. They delineate 3 phases, or differentiative transitions, in the development of the exocrine pancreas, involving first, morphogenesis and specific gene expression, second, overt cytodifferentiation and synthesis of pancreas-specific gene products and third, the selective modulation of gene expression in the mature organ. They also raise the possibility of distinguishing oncogenic target cells in terms of their position of this scale of progressive differentiation.

Overall, the papers in this volume present good evidence that cancer is essentially a disturbance of cell differentiation and that the process is not necessarily irreversible. It is a book which will interest not only individuals involved in cancer research, but also chemists, molecular biologists, cell biologists and geneticists. Many of the papers are provocative and stimulating; few are dull, and between them they provide a very useful and comprehensive bibliography.

R. M. Hicks